# Relationships between self-reported dyspnea, health conditions and frailty among Brazilian community-dwelling older adults: a cross-sectional study

**DOI:** 10.1590/1516-3180.2021.0237.R2.27072021

**Published:** 2022-03-18

**Authors:** Giselle Layse Andrade Buarque, Flávia Silva Arbex Borim, Anita Liberalesso Neri, Mônica Sanches Yassuda, Ruth Caldeira de Melo

**Affiliations:** I PT, MSc. Physiotherapist and Doctoral Student, Postgraduate Program on Gerontology, Faculty of Medical Sciences, Universidade Estadual de Campinas (UNICAMP), Campinas (SP), Brazil.; II PT, PhD. Physiotherapist, Assistant Professor, Department of Collective Health, School of Health Sciences, Universidade de Brasília (UnB), Brasília (DF), Brazil; and Advisor, Postgraduate Program on Gerontology, Faculty of Medical Sciences, Universidade Estadual de Campinas (UNICAMP), Campinas (SP), Brazil.; III PhD. Psychologist and Collaborating Professor, Department of Medical Psychology and Psychiatry and Advisor, Postgraduate Program on Gerontology, Faculty of Medical Sciences, Universidade Estadual de Campinas (UNICAMP), Campinas (SP), Brazil.; IV PhD. Psychologist, Full Professor, School of Arts, Sciences and Humanities, and Advisor, Postgraduate Program on Gerontology, School of Arts, Sciences and Humanities, Universidade de São Paulo (USP), São Paulo (SP), Brazil; and Advisor, Postgraduate Program on Gerontology, Universidade Estadual de Campinas (UNICAMP), Campinas (SP), Brazil.; V PT, PhD. Physiotherapist and Assistant Professor, School of Arts, Sciences and Humanities, and Advisor, Postgraduate Program on Gerontology, School of Arts, Sciences and Humanities, Universidade de São Paulo (USP), São Paulo (SP), Brazil.

**Keywords:** Aged, Dyspnea, Frailty, Elderly, Breathlessness, Noncommunicable diseases, Exhaustion

## Abstract

**CONTEXT:**

Dyspnea is a symptom present in several chronic diseases commonly seen among older adults. Since individuals with dyspnea tend to stay at rest, with consequently reduced levels of physical activity, they are likely to be at greater risk of developing frailty, especially at older ages.

**DESIGN AND SETTING:**

Cross-sectional study at community level, Brazil.

**OBJECTIVE:**

To analyze the relationships between self-reported dyspnea, health conditions and frailty status in a sample of community-dwelling older adults.

**METHOD:**

Secondary data from the follow-up of the Frailty in Brazilian Elderly (FIBRA) study, involving 415 community-dwelling older adults (mean age: 80.3 ± 4.68 years), were used. The variables analyzed were sociodemographic characteristics, reported dyspnea, clinical data and frailty phenotype. Associations between dyspnea and other variables (age, sex, education and body mass index) were verified through the crude (c) and adjusted (a) odds ratios.

**RESULTS:**

The prevalence of dyspnea in the entire sample was 21.0%. Dyspnea was more present in individuals with pulmonary diseases, heart disease, cancer and depression. Older adults with multimorbidities (adjusted odds ratio, ORa = 2.91; 95% confidence interval, CI = 1.41-5.99) and polypharmacy (ORa = 2.02; 95% CI = 1.15-3.54) were more likely to have dyspnea. Those who reported dyspnea were 2.54 times more likely to be frail (ORa = 2.54; 95% CI = 1.08-5.97), and fatigue was their most prevalent phenotype component.

**CONCLUSION:**

Dyspnea was associated with different diseases, multimorbidities, polypharmacy and frailty. Recognizing the factors associated with dyspnea may contribute to its early identification and prevention of its negative outcomes among older adults.

## INTRODUCTION

Dyspnea is a symptom that is present in different chronic diseases, with high prevalence among older adults.^1,2^ The American Thoracic Society defines dyspnea as “a subjective experience of breathing discomfort that consists of qualitatively distinct sensations that vary in intensity”.^3^ Since dyspnea is a frequent symptom among community-dwelling older adults,^2,4^ it is important that healthcare professionals should routinely evaluate and document the presence of dyspnea, just like they do for pain.^3,5^

In clinical practice, dyspnea is initially assessed by a single question (e.g. “Are you short of breath? Yes or No).^6^ Single-item questionnaires have clinical importance, as they are simple and quick to use, but they fail to cover all aspects of dyspnea.^7^ A large number of scales for measuring dyspnea have been described in the literature. Scales based on reports of breathlessness during activities of daily living (e.g. the modified Medical Research Council Dyspnea Scale and the Baseline Dyspnea Index) and on the perception of breathlessness while making exertions (e.g. the modified Borg scale and visual analogue scale) are the ones most frequently used.^8^ Because multidimensional tools such as the Multidimensional Dyspnea Profile have the ability to assess different aspects of dyspnea, they are promising tools for use in both laboratory and clinical settings.^9^ Nonetheless, most breathlessness scales were developed and validated for patients with respiratory diseases and, consequently, they need to be properly tested before being applied to other populations.^7^

A systematic review showed that the estimated prevalence of dyspnea among community-dwelling older adults was 36%, and that dyspnea can have an impact on different activities of daily living.^2^ In Brazil, a cross-sectional study among community-dwelling older adults observed that 30.9% of the participants had dyspnea symptoms. Moreover, most of the older adults who reported dyspnea had poor physical performance, were women, had multimorbidity and were frail.^4^ In addition to presenting an association between dyspnea and cardiorespiratory diseases, older adults with moderate to severe dyspnea are more likely to be unable to perform a single chair stand, have depressive symptoms, use psychoactive drugs and be underweight and/or obese, than those with no or mild dyspnea.^1,10^ Because presence of dyspnea is associated with several chronic diseases and health issues, some authors have argued that dyspnea in older adults should be considered to be a multifactorial geriatric condition.^10,11^

Common adaptive responses to the presence of dyspnea include avoidance of physical effort and, consequently, adoption of a sedentary lifestyle.^3^ In patients with chronic obstructive pulmonary disease (COPD), this can precipitate a downward spiral of dyspnea-induced inactivity, thus resulting in physical deconditioning, progression of disease and disability.^12^ Thus, some studies have shown that COPD patients were more likely to be frail, and that this was associated with airflow limitations, reduced physical function, dyspnea, disability, anxiety and depression.^13^

Frailty is a medical syndrome with multiple causes and contributors that confers vulnerability to stressors and increases the risk of different negative outcomes at older age, such as dependency and/or death.^14^ Regarding physical frailty, physical inactivity plays an important role in the progression of this syndrome, as it directly influences some determinant components for development of frailty.^15^ Although the overlap between dyspnea-induced inactivity and vicious cycles of frailty seems obvious, the relationships between dyspnea, health conditions and frailty among community-dwelling older adults are not well established.^16^ Vaz Fragoso et al.,^16^ for example, found that older adults with respiratory impairment had higher chances of becoming frail, and vice versa. In another study,^17^ the same authors observed that poor physical-functional performance and frailty status were associated with moderate to severe exertional dyspnea in community-dwelling older adults.

Considering that the older population is growing and that dyspnea is a highly prevalent symptom in this phase of life, it would be useful to identify the factors associated with this important symptom, especially with a view to adequate healthcare management and/or prevention of negative outcomes among older adults. In addition, in the presence of dyspnea, the person tends to stay at rest, which can cause a series of negative consequences, such as physical inactivity, deconditioning and disability, which are commonly associated with the onset and progression of frailty syndrome.

## OBJECTIVE

In order to collaborate in healthcare practices for older adults, the aim of the present study was to analyze the relationships between self-reported dyspnea, health conditions and the frailty phenotype in a sample of community-dwelling older adults.

## METHODS

### Study design and participants

This was a cross-sectional study that used the records of community-dwelling older adults who participated in the second wave of the Frailty in Brazilian Elderly (FIBRA) study, conducted in the city of Campinas and in the Ermelino Matarazzo district of the city of São Paulo, state of São Paulo, Brazil, between 2016 and 2017. The first wave of data collection occurred between 2008 and 2009 and its database contained a record of 1284 older adults. Details about the sample size calculation, recruitment, data collection and main findings of the first wave can be found elsewhere.^18^

The second wave of the FIBRA study was submitted to and approved by the local ethics committees on November 23, 2015 (approval number, CAAE: 49987615.3.0000.5404) and on September 17, 2018 (CAAE: 92684517.5.0000.5404). The present study was also approved by the ethics committee board on October 31, 2019 (CAAE: 22196619.7.0000.5390).

### Procedures

The participants in the first wave of the FIBRA study were contacted and invited to participate in the follow-up study. At the beginning of the single session of data collection, which was conducted at the participants’ homes, these older adults were informed about study procedures and voluntarily signed an informed consent statement, after agreeing to participate.

Older adults who fulfilled the inclusion and exclusion criteria completed the interview. The inclusion criteria were as follows: the participants needed to have participated in the first wave and to have remained as permanent residents in the geographical location. Older adults with cognitive impairment suggestive of dementia, severe disability (wheelchair-bound or bedridden), motor and/or cognitive sequelae from stroke, moderate/severe Parkinson’s disease, severe visual and/or hearing impairments, or decompensated diseases or terminal illness, were excluded. Considering that the number of chronic diseases (cardiovascular, respiratory, cancerous, etc.) increases with age,^19^ older adults with these diseases were not excluded from the study. In addition, given that information on comorbidities was accessed through self-reporting (detailed below) and access to medical files was not feasible, it was not possible to classify the diseases reported with exactness (e.g. the type of respiratory disease reported). Current smokers were also included in the study since their prevalence in the total sample was low (i.e. 3.0%) (Table 1).

**Table 1.** Percentage distribution of the sample and the proportion of dyspnea according to sociodemographic variables, anthropometric data, health conditions, and frailty. FIBRA study, Campinas and Ermelino Matarazzo district in São Paulo, São Paulo, Brazil, 2016/2017

Variables	n (%)	Dyspnea		P-value
		No n (%)	Yes n (%)	
**Sex**				
Male	125 (30.2)	105 (84.0)	20 (16.0)	0.100
Female	289 (69.8)	222 (76.8)	67 (23.2)	
**Age groups (years)**				
72-79	180 (43.6)	132 (73.3)	48 (26.7)	**0.014**
≥ 80	233 (56.4)	194 (83.3)	39 (16.7)	
**Schooling level**				
Illiterate	52 (13.3)	38 (73.1)	14 (26.9)	0.398
1-4 years	234 (59.7)	188 (80.3)	46 (19.7)	
≥ 5 years	106 (27.0)	87 (82.1)	19 (17.9)	
**Body mass index classification**				
Underweight	72 (17.3)	62 (86.1)	10 (13.9)	
Normal	180 (43.8)	147 (81.7)	33 (18.3)	0.075
Overweight	50 (12.0)	38 (76.0)	12 (24.0)	
Obese	113 (27.2)	81 (71.7)	32 (28.3)	
**Current smoker**				
Yes	12 (3.0)	9 (75.0)	3 (25.0)	0.985
No	399 (97.0)	306 (79.1)	81 (20.9)	
**Polypharmacy**				
0-4	219 (62.6)	184 (84.0)	35 (16.0)	**0.002**
≥ 5	131 (37.4)	92 (70.2)	39 (29.8)	
**Multimorbidity**				
0-1	118 (30.7)	106 (89.8)	12 (10.2)	**0.002**
≥ 2	267 (69.3)	203 (76.0)	64 (24.0)	
**Frailty**				
Not frail	93 (22.6)	77 (82.8)	16 (17.2)	**0.022**
Pre-frail	265 (64.3)	213 (80.4)	52 (19.6)	
Frail	54 (13.1)	35 (64.8)	19 (35.2)	

From the original cohort, 549 older adults (≥ 65 years of age) agreed to participate in the second wave. Another 192 had died, and 543 could not located or refused to participate. Among those who completed the initial part of the interview (sociodemographic information, anthropometric measurements, physical frailty assessment and Mini-Mental State Examination, MMSE), 419 had MMSE scores above the cutoff point, adjusted for years of schooling, and participated in the second block of measurements. Out of these, 415 participants answered the question about the presence of dyspnea (i.e. the dependent variable) and thus were included in the present study.

It is important to note that a sample size ≥ 329 participants was found to be adequate for the present study, based on an estimated prevalence of dyspnea of 30.9%,^4^ a 95% confidence level and an error of 0.5%.

The following variables/scales were extracted from the FIBRA study database:

- Self-reported dyspnea: The participants were asked about the presence of dyspnea through a simple question: “Do you have breathlessness, yes or no?”.
- Sociodemographic variables: Sex (men, women); age ranges (72-79 years and ≥ 80 years); and schooling (illiterate, 1-4 years and ≥ 5 years).
- Anthropometric data: Weight was measured in kilograms, using the G-Tech brand scale; and height in centimeters, using a scale (200 cm), graduated in centimeters and millimeters. Body mass index (BMI) was obtained by dividing the weight (kg) by the height squared (m^2^). BMI was classified in accordance with the cutoff values established by the Pan-American Health Organization (PAHO). In this way, older adults are categorized as follows: underweight with BMI ≤ 23 kg/m^2^; normal weight with BMI between > 23 kg/m^2^ and < 28 kg/m^2^; overweight with BMI between > 28 kg/m^2^ and < 30 kg/m^2^; and obese ≥ 30 kg/m^2^.^20^
- Smoking habit: Participants were considered to be current smokers if they answered “yes” to the following question: “Do you currently smoke?”.
- Self-reported chronic diseases: Information was obtained from dichotomous items (yes or no) in which it was investigated whether a doctor had diagnosed heart diseases, hypertension, diabetes mellitus, depression, pulmonary diseases, osteoporosis, stroke, cancer and/or osteoarthritis at any time during the 12 months prior to the interview. Multimorbidity was considered to be the coexistence of two or more of these diseases.
- Frailty: This was assessed based on the phenotype criteria proposed by Fried et al.^15^ Older adults were qualified as frail (three or more criteria), pre-frail (one or two criteria) or non-frail (no criteria), considering the following five criteria:
- Self-reported unintentional weight loss: this was considered to be present if, over the last year, it was greater than or equal to 4.5 kg or 5% of body weight.
- Fatigue: this was considered to be present if the older adults answered “occasionally” or “most of the time” for either of the following two self-report items regarding how they felt during the last week: “I felt that everything I did was an effort” and “I could not get going”. These items were obtained from the Center for Epidemiologic Studies Depression Scale (CESD).^21,22^
- Low grip strength of the dominant hand: this was considered to be present, as measured using a Jamar dynamometer (Lafayette Instruments, Lafayette, Indiana, United States), if the average measurement from three grip attempts with the arm flexed at 90° at the forearm was among the 20% lowest distribution values, after adjusting for sex and BMI.
- Gait slowness: this was considered to be present, as assessed from the time in seconds spent to walk a distance of 4.0 meters at the usual speed, if the average of three attempts was among the 20% highest distribution values (in seconds) for the entire sample, after adjusting for sex and height.^23^
- Inactive level of physical activity: The level of physical activity corresponded to the weekly frequency and daily duration of physical exercise, sports and household chores, based on responses to the items of the Minnesota Leisure Time Activity Questionnaire.^24^ To calculate the weekly caloric expenditure on leisure activities and on household chores, we considered the number of items to which the older adult replied affirmatively, multiplied by the number of days in the week and the number of minutes per day. Then we calculated the quintiles of the distribution of this variable, for men and women separately. We considered participants to be inactive if they scored among the 20% lowest distribution values for weekly caloric expenditure, corrected according to sex.^25^

### Statistical analysis

The sample was characterized by calculating the absolute and relative frequencies and 95% confidence interval (95% CI) of the variables considered. Associations between dyspnea and the other variables were verified using Pearson’s chi-square test or Fisher’s exact test. After that, simple and multiple logistic regression analyses were also used to estimate crude and adjusted odds ratios. The adjusted analysis was performed independently according to age, sex, education and BMI classifications for each variable. For all analyses, the significance level was set at P < 0.05.

## RESULTS

Four hundred and fifteen older people (mean age 80.3 ± 4.68 years old) answered the question about shortness of breath. The majority of the participants were very old (56.4% were aged 80 years and over), were females (69.8%), had had one to four years of education (59.7%), had normal weight (i.e. 43.8% had BMI between > 23 kg/m^2^ and < 28 kg/m^2^) and were not currently smokers (97.0%). In addition, these older adults mostly had two or more self-reported diseases (69.3%), used less than five medications (62.6%) and were classified as pre-frail (64.3%) (Table 1).

The prevalence of dyspnea in the entire sample was 21% (95% CI = 17.3 to 25.2). The frequencies and associations between dyspnea and sociodemographic variables, anthropometric data, health conditions and frailty are presented in Table 1. No significant difference between the sexes was found with regard to the prevalence of dyspnea, but the prevalence was greater (P = 0.014) in the younger age group (26.7% for 72-79 years of age) than in the older group (16.7% for 80 years and over). However, presence of dyspnea was not associated with BMI (P = 0.075) or with the smoking habit (P = 0.985).

**Figure 1.**
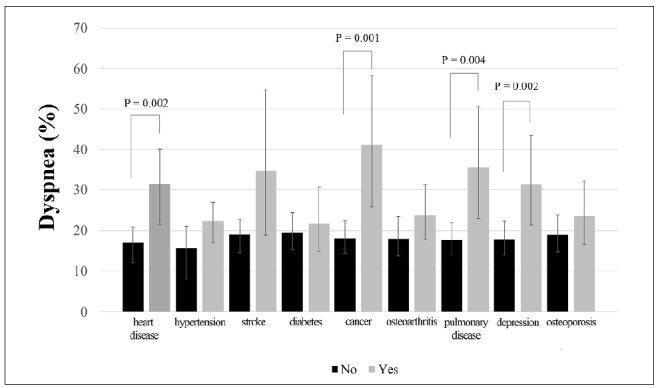
Prevalence of dyspnea according to self-reported diseases.

**Figure 2.**
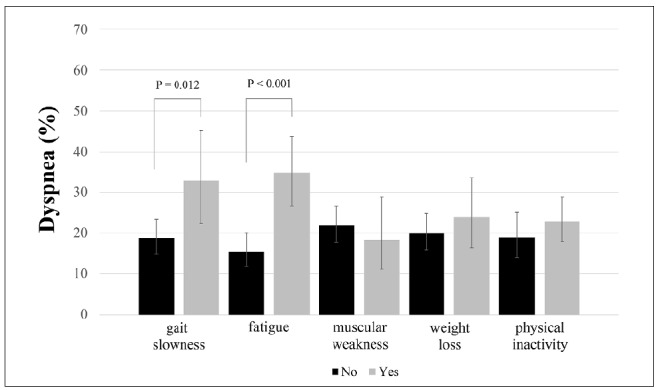
Prevalence of dyspnea according to the presence of frailty phenotype components.

In relation to health conditions, dyspnea was also more prevalent among participants with polypharmacy (29.8% versus 16.0%; P = 0.002) and multimorbidities (24.0% versus 10.2%; P = 0.002). Figure 1 shows the prevalence of dyspnea according to the self-reported diseases evaluated in the present study. Dyspnea was more frequent among older adults with heart diseases (31.5% versus 17.0%; P = 0.002), cancer (41.2% versus 18.1%; P = 0.001), pulmonary diseases (35.5% versus 17.6%; P = 0.004) and depression (31.3% versus 17.8%; P = 0.012). In addition, the prevalence of dyspnea was higher (P = 0.022) in older adults who were frail (35.2%) than in those classified as pre-frail (19.6%) or non-frail (17.2%) (Table 1). The analysis on frailty components showed that dyspnea was more present in older adults who presented fatigue (34.7% versus 15.4%; P = 0.000) and gait slowness (32.8% versus 18.8%; P = 0.012) (Figure 2).

After adjusting for age, sex, schooling and BMI classification, the logistic regression showed that the participants with pulmonary diseases (adjusted odds ratio, ORa = 2.25; 95% CI = 1.08 to 4.67), heart diseases (ORa = 2.06; 95% CI= 1.17 to 3.62), cancer (ORa = 3.25; 95% CI = 1.48 to 7.18) and depression (ORa = 1.94; 95% CI = 1.04 to 3.59) were more likely to have dyspnea. Other health conditions also associated with the presence of dyspnea were multimorbidities (ORa = 2.91; 95% CI = 1.41 to 5.99) and polypharmacy (ORa = 2.02; 95% CI = 1.15 to 3.54). Moreover, older adults who reported dyspnea had 2.54 times (95% CI = 1.08 to 5.97) more chances of being classified as frail than as pre-frail or non-frail (Table 2).

**Table 2.** Crude and adjusted odds ratios for dyspnea, according to sociodemographic and anthropometric characteristics, health and clinical conditions, and frailty status and phenotype components of the FIBRA study. City of Campinas and Ermelino Matarazzo district of the city of São Paulo, São Paulo, Brazil, 2016/2017

Variables	Dyspnea					
	OR_crude_	95% CI	P-value	OR_adjusted_	95% CI	P-value
**Sociodemographic and anthropometric characteristics**						
**Sex**						
Female versus male	1.58	0.91-2.75	0.101	1.37	0.77-2.44	0.439
**Age groups (years)**						
≥ 80 versus 72-79	0.55	0.34-0.89	**0.015**	0.59	0.35-0.98	**0.041**
**Schooling level**						
1-4 years versus illiterate	0.66	0.33-1.33	0.247	0.64	0.31-1.32	0.228
≥ 5 years versus illiterate	0.59	0.27-1.30	0.194	0.56	0.26-1.24	0.209
**Body mass index classification**						
Underweight versus normal	0.72	0.33-1.55	0.398	0.64	0.27-1.49	0.301
Overweight versus normal	1.41	0.66-2.98	0.373	1.39	0.63-3.08	0.412
Obese versus normal	1.76	1.01-3.07	**0.047**	1.54	0.86-2.78	0.148
**Health and clinical conditions**						
**Self-reported diseases**						
Heart diseases	2.24	1.32-3.80	**0.003**	2.06	1.17-3.62	**0.012**
Hypertension	1.55	0.88-2.73	0.128	1.53	0.84-2.82	0.167
Stroke	2.25	0.96-5.27	0.061	1.50	0.58-3.86	0.399
Diabetes	1.14	0.66-1.98	0.620	1.10	0.62-1.95	0.746
Cancer	3.17	1.52-6.59	**0.002**	3.25	1.48-7.18	**0.003**
Arthritis	1.42	0.86-2.34	0.164	1.41	0.82-2.40	0.211
Pulmonary diseases	2.57	1.31-5.03	**0.006**	2.25	1.08-4.67	**0.029**
Depression	2.11	1.17-3.80	**0.013**	1.94	1.04-3.59	**0.036**
Osteoporosis	1.31	0.77-2.22	0.308	1.41	0.79-2.50	0.239
**Multimorbidity**						
≥ 2 versus 0-1	2.78	1.44-5.39	**0.002**	2.91	1.41-5.99	**0.004**
**Polypharmacy**						
≥ 5 versus 0-4	2.23	1.32-3.75	**0.003**	2.02	1.15-3.54	**0.014**
**Frailty status and phenotype components**						
**Frailty**						
Pre-frail versus not frail	1.17	0.63-2.18	0.609	1.14	0.58-2.22	0.705
Frail versus not frail	2.61	1.20-5.67	**0.015**	2.54	1.08-5.97	**0.032**
**Frailty components (versus absence of the criterion)**						
Weight loss	1.26	0.73-2.18	0.398	1.48	0.81-2.69	0.197
Low physical activity	1.26	0.78-2.04	0.330	1. 20	0.72-2.03	0.482
Low grip strength	0.80	0.42-1.52	0.509	0.81	0.40-1.64	0.563
Gait slowness	2.10	1.16-3.79	**0.013**	1.71	0.89-3.30	0.106
Fatigue	2.90	1.77-4.75	**< 0.001**	2.75	1.62-4.66	**< 0.001**

OR = odds ratio; CI = confidence interval; adjusted: age, sex, schooling and body mass index classification. In bold: statistically significant associations.

Among the frailty phenotype components, fatigue (crude odds ratio, ORc = 2.90; 95% CI = 1.77 to 4.75) and gait slowness (ORc = 2.10; 95% CI = 1.16 to 3.79) showed associations with dyspnea in the crude analysis. After controlling for confounding variables (i.e. age, sex, schooling and BMI classification), only fatigue remained significantly associated with dyspnea (ORa = 2.75; 95% CI = 1.62 to 4.66).

**Figure 3.**
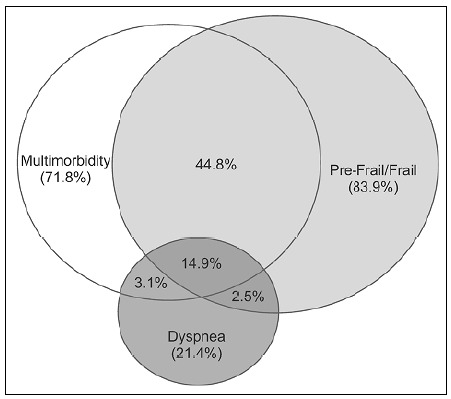
Overlapping between dyspnea, multimorbidity and frailty/pre-frailty status.

Figure 3 shows the overlaps between the presences of dyspnea, multimorbidity and frailty/pre-frailty. The main overlapping detected was between multimorbidity and frailty (44.8%), followed by dyspnea versus multimorbidity versus frailty/pre-frailty (14.5%). The rates of overlapping of dyspnea with multimorbidity and frailty/pre-frailty were 3.1% and 2.5%, respectively. In addition, dyspnea only appeared separately from multimorbidity and frailty in fewer than 1% of the participants. It is important to note that 7.3% of the participants did not have any of these conditions, either alone or in combination.

## DISCUSSION

In the present study, the prevalence of dyspnea among community-dwelling older adults was 21%, which was within the range reported by previous studies, even those differing in assessment method used (i.e. single question versus the Medical Research Council Dyspnea Scale).^2,4^ Older adults who reported having breathlessness were more likely to have pulmonary diseases, heart diseases, cancer, depression, multimorbidity and polypharmacy. In addition, dyspnea occurred more frequently among participants who were classified as frail (i.e. 35.2%), and fatigue was the phenotype component that was most strongly associated with dyspnea.

There is evidence in the literature that the prevalence of dyspnea increases with advancing age.^2,26,27^ In a representative sample of North American older adults,^1^ it was observed that one in four adults aged 70 and over experienced clinically significant breathlessness, evaluated via a single question (i.e. “How often do you become short of breath while awake?”). Greater prevalence of multimorbidities^10^ and polypharmacy,^28^ reduced physical fitness^3^ and decreased efficiency of the respiratory system^11^ make older adults more susceptible to dyspnea, which corroborate the increasing prevalence of dyspnea among older age ranges.

Although our results were in agreement with the literature in terms of the overall prevalence of dyspnea among older adults, it was surprisingly higher among those aged 72 to 79 years than among those aged 80 and over. Nonetheless, a previously published geriatric dyspnea model supports the notion of paradoxical reduction in the perception and reporting of dyspnea, explained in terms of the influence of physiological, neurological, psychological and social changes with aging.^29^ Thus, it is plausible that the self-reported dyspnea rate is reduced in the older age ranges. Additionally, another study with the same cohort showed that a considerable portion of longer-living older adults (i.e. 80 years of age and over) were in good health,^30^ which contradicted the results from investigations in other countries.^31,32,33^ One hypothesis is that people with better health and socioeconomic conditions are more likely to reach advanced ages. So, it is possible that the lower prevalence of dyspnea in the oldest-old group can also be partly due to this (i.e. older adults with worse conditions, including those associated with the presence of dyspnea, would die earlier).^34,35^ Given that cohort studies involving very old community-dwelling adults are scarce in Brazil, the possibility for discussion of the present results is limited.

Regarding the factors associated with dyspnea in older adults, the present results partly corroborate the findings of Smith et al.^1^ Among community-dwelling older adults, those authors found that individuals with respiratory diseases, multimorbidity, heart diseases, obesity and low educational level were more likely to have dyspnea. In addition, dyspnea was associated with depression, anxiety, fatigue, pain, dependence with regard to activities of daily living and high use of healthcare services. Interestingly, Johnson et al.^36^ showed that age was inversely related to restrictive breathlessness during the last year of life. They argued that individuals who lived to old age achieved that age because they probably did not have the medical conditions associated with restrictive dyspnea, which thus supports the hypothesis raised above.

In old age, the presence of multimorbidity may be more complex due to overlapping of other conditions such as physical impairment, mental disorders, frailty and polypharmacy.^30,37,38^ These conditions impose a considerable burden at an individual level, and on healthcare and social services too.^33^ Previous studies demonstrating the complexity of healthcare management for older people showed that multimorbidity and polypharmacy were also associated with dyspnea,^1,28^ which was concordant with our findings. Smith et al.,^1^ for example, observed that older adults with significant breathlessness were more likely to have multimorbidity than single chronic conditions.

A cross-sectional study conducted in Brazil also showed that community-dwelling older adults with dyspnea, assessed using the modified Medical Research Council Dyspnea Scale, had more diseases than those without this symptom.^4^ Regarding polypharmacy, Akgün et al.^28^ observed that it was strongly related to dyspnea (as measured on a shortness-of-breath scale from 0 to 10 scale within the Edmonton Symptom Assessment System) among older adults with serious life-limiting diseases. After adjusting for different factors (age, sex, diagnosis and statin discontinuation), each additional medication was associated with 8% and 16% increased risk of mild and moderate-to-severe dyspnea, respectively. In addition to associations between dyspnea and some chronic self-reported diseases, multimorbidity and polypharmacy, it was also observed in the present study that dyspnea could occur in combination with multimorbidity and/or frailty/pre-frailty conditions. These, in turn, may bring more complexity to healthcare for community-dwelling older adults.

The relationship between respiratory impairment/diseases and frailty among older adults has been demonstrated in different studies.^13,16,39^ In a meta-analysis, Marengoni et al.^39^ found that older adults with COPD had twofold increased odds for presenting frailty, compared with those without COPD. Among community-dwelling older adults, Vaz Fragoso et al.^16^ showed that respiratory impairment was associated with frailty at the baseline and that there was an increased likelihood of developing frailty features after three years of follow-up. Although studies involving COPD patients have supported the existence of a relationship between dyspnea and frailty,^13,39,40^ it is not known whether this is true for community-dwelling older adults.

In the present study, older adults who reported dyspnea were more likely to be classified as frail, even after adjustment for age, sex, schooling and BMI classification. Moreover, although fatigue and gait slowness were the frailty phenotype components that were associated with reports of dyspnea, only fatigue remained associated with dyspnea after controlling for confounding factors. According to Vaz Fragoso et al.,^17^ poor performance in the single chair stand was associated with a greater chance of having moderate-severe exertional dyspnea, which became attenuated when controlled for some frailty components singly. Similarly, Silva et al.^4^ observed that there was high prevalence of frailty (23.6%) among Brazilian community-dwelling older adults who reported having dyspnea. After adjusting for different confounders, higher dyspnea score was found to be independently associated with poor physical performance.^4^ Among COPD patients, Medina-Mirapeix et al.^40^ demonstrated that low grip strength was the most prevalent component among those classified as frail, followed by low physical activity levels and fatigue. However, only fatigue was associated with dyspnea and severity of COPD symptoms in the final multivariate models.

Some conceptual models for explaining the dyspnea-inactivity vicious cycle in COPD are available in the literature. Recently, Ramon et al.^41^ proposed a model in which a sequence of events (e.g. expiratory airflow limitation, increased resting lung volumes and dynamic hyperinflation) might lead to a cycle consisting of the following: dyspnea - reduced physical activity - deterioration of exercise capacity - dyspnea. Although not included in Ramon’s model, fatigue might be easily fit into the dyspnea-inactivity vicious cycle, independently of a specific diagnosis. In an observational study involving community-dwelling older adults, Egerton et al.^42^ showed that fatigue was associated with lower physical activity levels. This finding did not confirm whether fatigue was a cause of low physical activity levels or whether it occurred as a result from inactivity. Therefore, those authors suggested that reduced activity might also lead to decreased physical capacity, which would hence increase inactivity-related fatigue through a reversed causal pathway. Surprisingly, neither low physical activity levels nor low grip strength was associated with dyspnea in our study. Therefore, it is still unclear whether dyspnea-inactivity and frailty cycles overlap or not among community-dwelling older adults and whether a reversed causal pathway might exist in this population. This pathway would consist of the following: frailty - reduced physical activity - deterioration of physical reserves and increased exercise intolerance - dyspnea.

In summary, it was observed that a considerable proportion of the community-dwelling older adults of our study reported shortness of breath. Unlike other age groups, this symptom may be related not only to specific conditions (such as respiratory and cardiovascular diseases) but also to the presence of multimorbidities, polypharmacy and frailty. Thus, self-reported dyspnea should be considered to be an important symptom among older adults and should not be attributed to age *per se*. In addition to single-item questionnaires, it is important that shortness of breath should be investigated in a broader manner in this population, especially taking into account its impact on activities of daily living. However, it needs to be borne in mind that the scales most used for assessing dyspnea were developed before publication of the Consensus of the American Thoracic Society.^3^ Thus, these scales cannot cover the entirety of the definition of dyspnea in terms of functional, sensory-perceptual and affective aspects. In this way, multidimensional scales to evaluate dyspnea should be also considered when assessing the geriatric population. It is noteworthy that, in addition to adequate clinical management of chronic diseases and usage of medications, care for older adults with dyspnea should be carried out in an interdisciplinary and integrated manner, as much as possible.

The present study had some limitations that should be addressed. First of all, the cross-sectional nature of the study did not allow us to draw causal associations between dyspnea, health conditions and frailty. Therefore, further longitudinal studies should include objective respiratory measurements that would help clarify such relationships and the effect of respiratory impairment on the progression of frailty, especially among community-dwelling older adults. Second, as the present sample was not representative of the community-dwelling older population and only included individuals aged 72 years and over, the results need to be interpreted with caution. Third, as the presence of dyspnea was evaluated just by a single question, the impact of its severity, commonly assessed using the Medical Research Council Dyspnea Scale, was unknown in our sample. In addition, methodological bias cannot be ruled out, given that different approaches were used to measure dyspnea (a single screening question) and frailty (a well-established tool). However, it is important to note that no reliable and valid dyspnea scales for Brazilian community-dwelling older adults were available at the time of design and application of the research protocol.

## CONCLUSIONS

Among community-dwelling older adults, dyspnea was associated with different diseases, multimorbidities, polypharmacy and frailty, even after controlling for age, sex, schooling and body mass index. Among those reporting dyspnea, fatigue and gait slowness were more likely to be present. Recognizing the factors associated with reports of dyspnea may contribute to prevention of negative outcomes and to management of frailty in this population.

## References

[1] Smith AK, Currow DC, Abernethy AP (2016). Prevalence and Outcomes of Breathlessness in Older Adults: A National Population Study. J Am Geriatr Soc..

[2] van Mourik Y, Rutten FH, Moons KG (2014). Prevalence and underlying causes of dyspnoea in older people: a systematic review. Age Ageing..

[3] Parshall MB, Schwartzstein RM, Adams L (2012). An official American Thoracic Society statement: update on the mechanisms, assessment, and management of dyspnea. Am J Respir Crit Care Med..

[4] Silva CFR, Pegorari MS, Matos AP, Ohara DG (2020). Dyspnea is associated with poor physical performance among community-dwelling older adults: a population-based cross-sectional study. Sao Paulo Med J..

[5] Banzett RB, Schwartzstein RM (2015). Dyspnea: Don’t Just Look, Ask!. Am J Respir Crit Care Med..

[6] MacDonald S, Yates J, Lance R, Giganti N, Chepurko D (2005). Are you asking the right admission questions when assessing dyspnea?. Heart Lung..

[7] Yorke J, Savin C (2010). Evaluating tools that can be used to measure and manage breathlessness in chronic disease. Nurs Times..

[8] Crisafulli E, Clini EM (2010). Measures of dyspnea in pulmonary rehabilitation. Multidiscip Respir Med..

[9] Banzett RB, O’Donnell CR, Guilfoyle TE (2015). Multidimensional Dyspnea Profile: an instrument for clinical and laboratory research. Eur Respir J..

[10] Miner B, Tinetti ME, Van Ness PH (2016). Dyspnea in Community-Dwelling Older Persons: A Multifactorial Geriatric Health Condition. J Am Geriatr Soc..

[11] Mahler DA (2017). Evaluation of Dyspnea in the Elderly. Clin Geriatr Med..

[12] Marchetti N, Kaplan A (2018). Dyspnea and Hyperinflation in Chronic Obstructive Pulmonary Disease: Impact on Physical Activity. Cleve Clin J Med..

[13] Lahousse L, Ziere G, Verlinden VJ (2016). Risk of Frailty in Elderly With COPD: A Population-Based Study. J Gerontol A Biol Sci Med Sci..

[14] Morley JE, Vellas B, van Kan GA (2013). Frailty consensus: a call to action. J Am Med Dir Assoc..

[15] Fried LP, Tangen CM, Walston J (2001). Frailty in older adults: evidence for a phenotype. J Gerontol A Biol Sci Med Sci..

[16] Vaz Fragoso CA, Enright PL, McAvay G, Van Ness PH, Gill TM (2012). Frailty and respiratory impairment in older persons. Am J Med..

[17] Vaz Fragoso CA, Araujo K, Leo-Summers L, Van Ness PH (2015). Lower Extremity Proximal Muscle Function and Dyspnea in Older Persons. J Am Geriatr Soc..

[18] Neri AL, Yassuda MS, de Araújo LF (2013). Metodologia e perfil sociodemográfico, cognitivo e de fragilidade de idosos comunitários de sete cidades brasileiras: Estudo FIBRA. Cad Saude Publica..

[19] Nunes BP, Batista SRR, Andrade FB (2018). Multimorbidity: The Brazilian Longitudinal Study of Aging (ELSI-Brazil). Rev Saude Publica..

[20] División de Promoción y Protección de la Salud (2001). Organización Panamericana de la Salud. Encuesta multicentrica salud bienestar y envejecimiento (SABE) en América Latina: informe preliminar. In: XXXVI Reunión del Comité Asesor de Investigaciónes en Salud.

[21] Lewinsohn PM, Seeley JR, Roberts RE, Allen NB (1997). Center for Epidemiologic Studies Depression Scale (CES-D) as a screening instrument for depression among community-residing older adults. Psychol Aging..

[22] Batistoni SST, Néri AL, Cupertino AP (2010). Validade e confiabilidade da versão Brasileira da Center for Epidemiological Scale - Depression (CES-D) em idosos Brasileiros. Psico-USF..

[23] Guralnik JM, Ferrucci L, Simonsick EM, Salive ME, Wallace RB (1995). Lower-extremity function in persons over the age of 70 years as a predictor of subsequent disability. N Engl J Med..

[24] Taylor HL, Jacobs DR, Schucker B (1978). A questionnaire for the assessment of leisure time physical activities. J Chronic Dis..

[25] Ainsworth BE, Haskell WL, Whitt MC (2000). Compendium of physical activities: an update of activity codes and MET intensities. Med Sci Sports Exerc..

[26] Huijnen B, van der Horst F, van Amelsvoort L (2006). Dyspnea in elderly family practice patients. Occurrence, severity, quality of life and mortality over an 8-year period. Fam Pract..

[27] Ho SF, O’Mahony MS, Steward JA (2001). Dyspnoea and quality of life in older people at home. Age Ageing..

[28] Akgün KM, Krishnan S, Feder SL (2020). Polypharmacy Increases Risk of Dyspnea Among Adults With Serious, Life-Limiting Diseases. Am J Hosp Palliat Care..

[29] Petersen S, von Leupoldt A, Van den Bergh O (2014). Geriatric dyspnea: doing worse, feeling better. Ageing Res Rev..

[30] Pivetta NRS, Marincolo JCS, Neri AL (2020). Multimorbidity, frailty and functional disability in octogenarians: A structural equation analysis of relationship. Arch Gerontol Geriatr..

[31] Ibarra-Castillo C, Guisado-Clavero M, Violan-Fors C (2018). Survival in relation to multimorbidity patterns in older adults in primary care in Barcelona, Spain (2010-2014): a longitudinal study based on electronic health records. J Epidemiol Community Health.

[32] Wang XX, Lin WQ, Chen XJ (2017). Multimorbidity associated with functional independence among community-dwelling older people: a cross-sectional study in Southern China. Health Qual Life Outcomes..

[33] Yarnall AJ, Sayer AA, Clegg A (2017). New horizons in multimorbidity in older adults. Age Ageing..

[34] Rizzuto D, Melis RJF, Angleman S, Qiu C, Marengoni A (2017). Effect of Chronic Diseases and Multimorbidity on Survival and Functioning in Elderly Adults. J Am Geriatr Soc..

[35] Marshall A, Nazroo J, Tampubolon G, Vanhoutte B (2015). Cohort differences in the levels and trajectories of frailty among older people in England. J Epidemiol Community Health..

[36] Johnson MJ, Bland JM, Gahbauer EA (2016). Breathlessness in Elderly Adults During the Last Year of Life Sufficient to Restrict Activity: Prevalence, Pattern, and Associated Factors. J Am Geriatr Soc..

[37] Bekić S, Babič F, Filipčić I, Trtica Majnarić L (2019). Clustering of Mental and Physical Comorbidity and the Risk of Frailty in Patients Aged 60 Years or More in Primary Care. Med Sci Monit..

[38] Schöttker B, Saum KU, Muhlack DC (2017). Polypharmacy and mortality: new insights from a large cohort of older adults by detection of effect modification by multi-morbidity and comprehensive correction of confounding by indication. Eur J Clin Pharmacol..

[39] Marengoni A, Vetrano DL, Manes-Gravina E (2018). The Relationship Between COPD and Frailty: A Systematic Review and Meta-Analysis of Observational Studies. Chest..

[40] Medina-Mirapeix F, Bernabeu-Mora R, Giménez-Giménez LM (2018). Physical frailty characteristics have a differential impact on symptoms as measured by the CAT score: an observational study. Health Qual Life Outcomes..

[41] Ramon MA, Ter Riet G, Carsin AE (2018). The dyspnoea-inactivity vicious circle in COPD: development and external validation of a conceptual model. Eur Respir J..

[42] Egerton T, Chastin SF, Stensvold D, Helbostad JL (2016). Fatigue May Contribute to Reduced Physical Activity Among Older People: An Observational Study. J Gerontol A Biol Sci Med Sci..

